# Using Bayes factors for testing hypotheses about intervention effectiveness in addictions research

**DOI:** 10.1111/add.13501

**Published:** 2016-08-10

**Authors:** Emma Beard, Zoltan Dienes, Colin Muirhead, Robert West

**Affiliations:** ^1^ Research Department of Clinical, Educational and Health PsychologyUniversity College LondonLondonUK; ^2^ Department of Epidemiology and Public HealthUniversity College LondonLondonUK; ^3^ School of PsychologyUniversity of SussexBrightonUK; ^4^ Institute of Health and SocietyNewcastle UniversityNewcastle upon TyneUK

**Keywords:** Addiction, Bayes factors, Bayesian, hypothesis testing, non‐significant, RCT

## Abstract

**Background and Aims:**

It has been proposed that more use should be made of Bayes factors in hypothesis testing in addiction research. Bayes factors are the ratios of the likelihood of a specified hypothesis (e.g. an intervention effect within a given range) to another hypothesis (e.g. no effect). They are particularly important for differentiating lack of strong evidence for an effect and evidence for lack of an effect. This paper reviewed randomized trials reported in *Addiction* between January and June 2013 to assess how far Bayes factors might improve the interpretation of the data.

**Methods:**

Seventy‐five effect sizes and their standard errors were extracted from 12 trials. Seventy‐three per cent (*n* = 55) of these were non‐significant (i.e. *P* > 0.05). For each non‐significant finding a Bayes factor was calculated using a population effect derived from previous research. In sensitivity analyses, a further two Bayes factors were calculated assuming clinically meaningful and plausible ranges around this population effect.

**Results:**

Twenty per cent (*n* = 11) of the non‐significant Bayes factors were < ⅓ and 3.6% (*n* = 2) were > 3. The other 76.4% (*n* = 42) of Bayes factors were between ⅓ and 3. Of these, 26 were in the direction of there being an effect (Bayes factor > 1 and < 3); 12 tended to favour the hypothesis of no effect (Bayes factor < 1 and > ⅓); and for four there was no evidence either way (Bayes factor = 1). In sensitivity analyses, 13.3% of Bayes Factors were < ⅓ (*n* = 20), 62.7% (*n* = 94) were between ⅓ and 3 and 24.0% (*n* = 36) were > 3, showing good concordance with the main results.

**Conclusions:**

Use of Bayes factors when analysing data from randomized trials of interventions in addiction research can provide important information that would lead to more precise conclusions than are obtained typically using currently prevailing methods.

## Introduction

Bayesian statistical analyses are being used increasingly in addictions research, and it has been proposed that this trend should accelerate 1. One important component of Bayesian analysis is the calculation of Bayes factors, which overcome many of the problems of traditional frequentist statistics 2. One of these is the misinterpretation that *P*‐values can be used to make claims of ‘no effect’ 3, 4, 5. *P*‐values signal the extremeness of the data under the assumption of the null hypothesis and so only tell us the probability of a test statistic at least as extreme as the one observed 6. Thus, a *P* > 0.05 may reflect evidence for ‘no effect’ or data insensitivity, i.e. a failure to distinguish the null hypothesis from the alternative because, for example, the standard error (SE) is high.

Bayes factors are the ratio of the (average) likelihood of two hypotheses being correct given a set of data. When evaluating interventions, the two hypotheses are typically H_1_: that the intervention had a desired effect (for a given range of plausible sizes), or within a certain range, versus H_0_: that it had no effect. Thus, a Bayes factor is equivalent to a likelihood ratio 7 (averaged over different plausible effect sizes) and thus is often denoted as:

$$\text{Bayes Factor}=\frac{\text{likelihood of data given}\;H_{1}}{\text{likelihood of data given}\;H_{0}}=\frac{P\left(D\left|H_{1}\right.\right)}{P\left(D\left|H_{0}\right.\right)}$$

which simply represents the probability of the data (D) given the alternative hypothesis divided by the probability of the data given the null hypothesis.

The use of Bayes factors has become more feasible in recent years following the development of online calculators 8 and R code 9, 10. Conventional cut‐offs for the interpretation of Bayes factors depend typically upon those set by Jeffreys 2 in the 1930s, with a Bayes factor greater than 3, or else less than ⅓, representing sufficient evidence to be taken note of for the experimental and null hypotheses, respectively; while values between approximately ⅓ and 3 indicate that the data are insensitive (see Table 1).

**Table 1:** Jeffreys’ Bayes factor cut‐offs.

*Bayes factor*	*Interpretation*
> 100	Extreme evidence for the experimental hypothesis
30–100	Very strong evidence for the experimental hypothesis
10–30	Strong evidence for the experimental hypothesis
3–10	Moderate evidence for the experimental hypothesis
1–3	Anecdotal evidence for the experimental hypothesis
1	No evidence
⅓–1	Anecdotal evidence for the null hypothesis
⅓–1/10	Moderate evidence for the null hypothesis
1/10–1/30	Strong evidence for the null hypothesis
1/30–1/100	Very strong evidence for the null hypothesis
< 1/100	Extreme evidence for the null hypothesis

The original label for 3 < Bayes factor < 10 was ‘substantial evidence’. Lee & Wagenmakers changed it to moderate, as they thought the original label sounded too decisive 3, 11.

This paper uses a set of randomized trials in the field of addiction to examine whether, and in what way, the conclusions may have been different had the authors calculated Bayes factors in their analyses. This should be useful in future research to assess whether and when to use this form of analysis.

## Calculating Bayes factors

Several software packages are available including an online calculator developed by Zoltan Dienes (http://www.lifesci.sussex.ac.uk/home/Zoltan_Dienes/inference/Bayes.htm) and a modified version by John Christie using R code, which allows one to adjust the quality of the estimation 9, 10.

Both approaches require the specification of an expected effect size (i.e. a plausible range of predicted values based on previous studies, judgement or clinical significance), the published effect size (e.g. mean difference or log odds ratio) and standard error of this parameter. They also both assume that the sampling distribution of the parameter estimate is distributed normally (hence the need to use the natural logs of odds ratios). The natural log of the odds ratio is approximately normally distributed with known standard error given by $\sqrt{\frac{1}{A}+\frac{1}{B}+\frac{1}{C}+\frac{1}{D}}$ , where A is the number of individuals in the experimental condition with the outcome of interest, B is the number of individuals in the experimental condition without the outcome of interest and C and D reflect the number of individuals with and without the outcome of interest in the control condition respectively (i.e. odds ratio = (A/B)/(C/D)), provided that these numbers are not very small. For adjusted odds ratios, and/or where standard errors (SE) are not reported, 95% confidence intervals (CI) can be used to derive the standard error {i.e. [LN(upper confidence interval)–LN(lower confidence interval)]/3.92}.

In instances where the primary outcome measure is a continuous variable, SEs can be derived for mean differences or regression coefficients (β) either using the standard formula for the SE of mean difference, i.e. [(SD^2^_control_/*n*_Control_) + (SD^2^_experimental_/*n*_experimental_)]; or *t*‐test values using [mean difference (or β)/*t*‐test value]; or (3) 95% CI = {[LN(upper confidence interval)–LN(lower confidence interval)]/3.92}.

A worked example, using the calculator associated with Dienes, can be found in Supporting information, [Link].

Others have advocated alternative methods of computing Bayes factors, including the Jeffreys–Zellner–Siow (JZS) *t*‐test 4, 12, which can be implemented in R 13, 14 (see Dienes & McLatchie, submitted, for comparison). Moves have also been made towards full Bayesian modelling, which requires a much more advanced knowledge of R or specialist software packages, and is beyond the scope of the current paper (e.g. WinBUGS) 3, 11.

## Methods

Bayes factors were calculated for 12 randomized controlled trials published in the first six issues of *Addiction* in 2013 (between January and June). Effect sizes, SEs, *P*–values and the main conclusions drawn by the authors were extracted from the papers for both primary and main secondary outcomes. Studies are generally only powered to detect estimated differences between experimental and control groups for the primary outcome, and thus Bayes factors may be particularly useful for secondary analyses 15, 16. Concerns have been raised previously regarding the interpretation of non‐significant findings for sensitivity analyses 15, 16.

Adjusted effect sizes (where available) and those reported at the longest point of follow‐up were used. Bayes factors were calculated using the online calculator provided by Dienes 8 and the modified version using R code by Christie 9, 10. Predicted values for the effect size or population standard deviation (SD) were based on previous studies (see Table 2). Additional sensitivity analyses were run to assess the effect of using higher and lower values. The chosen range was based either on the reported CI of the predicted effect size selected from previous publications or, when not available, the opinion of the lead author as to what would be a plausible effect.

**Table 2:** Results, conclusions and corresponding Bayes factors for randomized controlled trials (RCTs) published in *Addiction* in the first six issues of 2013.

*Study*	*Intervention*	*Control*	*Participants*	*Outcome*	*Sample mean*	*Sample standard error*	*Significance p*	*Study conclusions for non‐significant findings*	*Expected effect size*	*Bayes factor: Dienes (Christie)* 8, 9, 10	*Interpretation of Bayes factor using Dienes* 8	*Interpretation of Bayes Factors using Jeffreys* 2
Kypri 19	Web based alcohol screening and brief intervention for reducing hazardous drinking among Maori university students	Screening only	6697 students aged 17–24	P: Frequency of alcohol consumption	RaR 0.89	0.04	0.01**	‘Web‐based screening and brief intervention reduced hazardous and harmful drinking among non‐help‐seeking Maori students’	RaR 0.91^c^	17.5 (17.5)	Evidence for experimental hypothesis (i.e. an effect)	Strong evidence for experimental hypothesis
									RaR 0.85Ω	16.0 (16.0)	Evidence for experimental hypothesis (i.e. an effect)	Strong evidence for experimental hypothesis
									RaR 0.97Ω	5.3 (5.3)	Evidence for experimental hypothesis (i.e. an effect)	Moderate evidence for experimental hypothesis
				P: Quantity of alcohol	RaR 0.92	0.04	0.04*	No mention of results > 0.05	RaR 0.96^a^	3.0 (3.0)	Evidence for experimental hypothesis (i.e. an effect)	Moderate evidence for experimental hypothesis
									RaR 0.91Ω	3.4 (3.4)	Evidence for experimental hypothesis (i.e. an effect)	Moderate evidence for experimental hypothesis
									RaR 0.99Ω	1.4 (1.4)	Evidence is insensitive	Anecdotal evidence for experimental hypothesis
				P: Volume of alcohol	RaR 0.78	0.06	< 0.001***		RaR 0.89^a^	261.6 (261.3)	Evidence for experimental hypothesis (i.e. an effect)	Extreme evidence for experimental hypothesis
									RaR 0.82Ω	475.0 (466.2)	Evidence for experimental hypothesis (i.e. an effect)	Extreme evidence for experimental hypothesis
									RaR 0.96Ω	13.2 (13.2)	Evidence for experimental hypothesis (i.e. an effect)	Moderate evidence for experimental hypothesis
				P: Academic Role Expectation and Alcohol Scale (AREAS)	RaR 0.81	0.08	0.01*		RaR 0.95^a^	3.9 (3.9)	Evidence for experimental hypothesis (i.e. an effect)	Moderate evidence for experimental hypothesis
									RaR 0.82Ω	13.1 (13.1)	Evidence for experimental hypothesis (i.e. an effect)	Moderate evidence for experimental hypothesis
									RaR 0.99Ω	1.3 (1.3)	Evidence is insensitive	Anecdotal evidence for experimental hypothesis
				S: Binge drinking	OR 0.80	0.12	0.06		OR 0.89^a^	3.2 (3.2)	Evidence for experimental hypothesis (i.e. an effect)	Moderate evidence for experimental hypothesis
									OR 0.65Ω	2.8 (2.8)	Evidence is insensitive	Anecdotal evidence for experimental hypothesis
									OR 0.99Ω	1.1 (1.1)	Evidence is insensitive	Anecdotal evidence for experimental hypothesis
				S: Heavy drinking	OR 0.65	0.15	< 0.001***		OR 0.55^a^	19.0 (19.0)	Evidence for experimental hypothesis (i.e. an effect)	Strong evidence for experimental hypothesis
									OR 0.38Ω	13.9 (13.9)	Evidence for experimental hypothesis (i.e. an effect)	Strong evidence for experimental hypothesis
									OR 0.80Ω	15.5 (15.5)	Evidence for experimental hypothesis (i.e. an effect)	Strong evidence for experimental hypothesis
Li 20	Methadone maintenance therapy (MMT) care intervention (with motivational interviewing)	Standard care	41 providers and 179 clients from six clinics	P: Provider client interaction	MD 4.82	2.23	0.033*	‘The MMT CARE intervention targeting providers in methadone maintenance clinics can improve providers’ treatment knowledge and their interaction with clients. The intervention can also reduce clients’ drug‐using behaviour through motivational interviewing sessions conducted by trained providers. . It is difficult to explain the unexpected findings in provider MMT knowledge and client drug avoidance self‐efficacy [long term]; this may be a result of the small sample size and the pilot nature of the study’	MD 4.65^b^	5.6 (5.6)	Evidence for experimental hypothesis (i.e. an effect)	Moderate evidence for experimental hypothesis
									MD 2.18Ω	4.2 (4.2)	Evidence for experimental hypothesis (i.e. an effect)	Moderate evidence for experimental hypothesis
									MD 7.01Ω	4.9 (4.9)	Evidence for experimental hypothesis (i.e. an effect)	Moderate evidence for experimental hypothesis
				P: MMT knowledge	MD 1.00	0.56	0.544		MD 4.65^b^	1.1 (1.1)	Evidence is insensitive	Anecdotal evidence for experimental hypothesis
									MD 2.18Ω	2.1 (2.1)	Evidence is insensitive	Anecdotal evidence for experimental hypothesis
									MD 7.01Ω	0.7 (0.7)	Evidence is insensitive	Anecdotal evidence for null hypothesis
				P: Perceived stigma	MD −1.87	2.31	0.421		MD −5.1^c^	0.8 (0.8)	Evidence is insensitive	Anecdotal evidence for null hypothesis
									MD −1.2Ω	1.2 (1.2)	Evidence is insensitive	Anecdotal evidence for experimental hypothesis
									MD −9.0Ω	0.5 (0.5)	Evidence is insensitive	Anecdotal evidence for null hypothesis
				P: Perceived client support	MD 1.82	0.65	0.006**	No mention of results >0.05	MD 4.65^b^	12.9 (12.9)	Evidence for experimental hypothesis (i.e. an effect)	Strong evidence for experimental hypothesis
									MD 2.18Ω	20.8 (20.8)	Evidence for experimental hypothesis (i.e. an effect)	Strong evidence for experimental hypothesis
									MD 7.01Ω	8.9 (8.9)	Evidence for experimental hypothesis (i.e. an effect)	Moderate evidence for experimental hypothesis
				P: Drug avoidance self‐efficacy	MD 1.25	1.24	0.312		MD 0.9^d^	1.4 (1.4)	Evidence is insensitive	Anecdotal evidence for experimental hypothesis
									MD 0.3Ω	1.2 (1.2)	Evidence is insensitive	Anecdotal evidence for experimental hypothesis
									MD 1.5Ω	1.4 (1.3)	Evidence is insensitive	Anecdotal evidence for experimental hypothesis
				P: Concurrent drug use	OR 0.36	0.59	0.084		OR 0.66^e^	2.3 (2.3)	Evidence is insensitive	Anecdotal evidence for experimental hypothesis
									OR 0.56Ω	2.7 (2.7)	Evidence is insensitive	Anecdotal evidence for experimental hypothesis
									OR 0.78Ω	1.7 (1.7)	Evidence is insensitive	Anecdotal evidence for experimental hypothesis
Ward 21	Behavioural support and nicotine replacement therapy (NRT)	Behavioural support	269 adults in four primary care clinics	P: 12 month prolonged abstinence	OR 0.51	0.50	0.182	‘Nicotine patches may not be effective in helping smokers in low‐income countries to stop when given as an adjunct to behavioural support. . Our results do not support the incremental value of providing NRT in addition to behavioural counselling’	OR 1.51^f^	1.8 (1.8)	Evidence is insensitive	Anecdotal evidence for experimental hypothesis
									OR 1.35Ω	1.6 (1.6)	Evidence is insensitive	Anecdotal evidence for experimental hypothesis
									OR 1.70Ω	1.1 (1.1)	Evidence is insensitive	Anecdotal evidence for experimental hypothesis
				S: 7‐day point prevalence abstinence	OR 0.69	0.32	> 0.05	‘Between‐group differences [for 12 month prolonged abstinence] were not statistically significant at follow‐up. . . No significant between‐group differences were found for seven‐day point prevalence abstinence’	OR 1.78^f^	1.4 (1.4)	Evidence is insensitive	Anecdotal evidence for experimental hypothesis
									OR 1.49Ω	1.5 (1.5)	Evidence is insensitive	Anecdotal evidence for experimental hypothesis
									OR 2.12Ω	1.2 (1.2)	Evidence is insensitive	Anecdotal evidence for experimental hypothesis
Borland 22	OnQ: An interactive text messaging program	Minimal intervention	3530 smokers interested in quitting	P: 6‐months sustained abstinence	OR 1.44	0.24	> 0.05	‘Smokers interested in quitting who were assigned randomly to an offer of wither the internet‐based support program and/or the intervention automated text‐messaging program had a non‐significantly greater odds of quitting for at least 6 months than those randomized to an offer of a single website. . we failed to find clear significant effects between the intervention and the control’	OR 1.50^g^	2.2 (2.2)	Evidence is insensitive	Anecdotal evidence for experimental hypothesis
									OR 1.20α	2.0 (2.0)	Evidence is insensitive	Anecdotal evidence for experimental hypothesis
									OR 1.80α	1.9 (1.9)	Evidence is insensitive	Anecdotal evidence for experimental hypothesis
				S: 7‐day point prevalence abstinence	OR 1.20	0.15	> 0.05		OR 1.50^g^	1.2 (1.2)	Evidence is insensitive	Anecdotal evidence for experimental hypothesis
									OR 1.20α	1.6 (1.6)	Evidence is insensitive	Anecdotal evidence for experimental hypothesis
									OR 1.80α	0.9 (0.9)	Evidence is insensitive	Anecdotal evidence for null hypothesis
				S: Quit attempt	OR 1.11	0.12	> 0.05		OR 1.50^g^	0.6 (0.6)	Evidence is insensitive	Anecdotal evidence for null hypothesis
									OR 1.20α	1.1 (1.1)	Evidence is insensitive	Anecdotal evidence for experimental hypothesis
									OR 1.80α	0.4 (0.4)	Evidence is insensitive	Anecdotal evidence for null hypothesis
	QuitCoach: Personalized tailored internet‐delivered advice program	Minimal intervention		P: 6‐months sustained abstinence	OR 1.40	0.24	> 0.05	‘There were no differences in the proportion who reported making a quit attempt by the 1‐month follow‐up. . . At the 7‐month follow up, 8.5% of the sample achieved 6‐month sustained abstinence. No significant differences were found by condition, but the control condition was numerically least successful’.	OR 1.50^g^	1.9 (1.9)	Evidence is insensitive	Anecdotal evidence for experimental hypothesis
									OR 1.20α	1.8 (1.8)	Evidence is insensitive	Anecdotal evidence for experimental hypothesis
									OR 1.80α	1.6 (1.6)	Evidence is insensitive	Anecdotal evidence for experimental hypothesis
				S: 7‐day point prevalence abstinence	OR 1.03	0.15	> 0.05		OR 1.50^g^	0.4 (0.4)	Evidence is insensitive	Anecdotal evidence for null hypothesis
									OR 1.20α	0.7 (0.7)	Evidence is insensitive	Anecdotal evidence for null hypothesis
									OR 1.80α	0.3 (0.3)	Evidence for null hypothesis (i.e. no effect)	Moderate evidence for null hypothesis
				S: Quit attempt	OR 0.91	0.12	> 0.05		OR 1.50^g^	0.6 (0.6)	Evidence is insensitive	Anecdotal evidence for null hypothesis
									OR 1.20α	1.0 (1.0)	Evidence is insensitive	No evidence
									OR 1.80α	0.4 (0.4)	Evidence is insensitive	Anecdotal evidence for null hypothesis
	Integration of onQ and QuitCoach	Minimal intervention		P: 6‐months sustained abstinence	OR 1.06	0.15	> 0.05		OR 1.92^g^	0.3 (0.3)	Evidence for null hypothesis (i.e. no effect)	Moderate evidence for null hypothesis
									OR 1.40α	0.6 (0.6)	Evidence is insensitive	Anecdotal evidence for null hypothesis
									OR 2.40α	0.2 (0.2)	Evidence for null hypothesis (i.e. no effect)	Moderate evidence for null hypothesis
				S: 7‐day point prevalence abstinence	OR 1.45	0.24	> 0.05		OR 1.92^g^	1.8 (1.8)	Evidence is insensitive	Anecdotal evidence for experimental hypothesis
									OR 1.40α	2.3 (2.3)	Evidence is insensitive	Anecdotal evidence for experimental hypothesis
									OR 2.40α	1.5 (1.5)	Evidence is insensitive	Anecdotal evidence for experimental hypothesis
				S: Quit attempt	OR 1.03	0.12	> 0.05		OR 1.92^g^	0.2 (0.2)	Evidence for null hypothesis (i.e. no effect)	Moderate evidence for null hypothesis
									OR 1.40α	0.4 (0.4)	Evidence is insensitive	Anecdotal evidence for null hypothesis
									OR 2.40α	0.2 (0.2)	Evidence for null hypothesis (i.e. no effect)	Moderate evidence for null hypothesis
	Choice of either alone or combined program	Minimal intervention		P: 6‐months sustained abstinence	OR 1.47	0.24	> 0.05		OR 1.92^g^	2.0 (2.0)	Evidence is insensitive	Anecdotal evidence for experimental hypothesis
									OR 1.40α	2.5 (2.5)	Evidence is insensitive	Anecdotal evidence for experimental hypothesis
									OR 2.40α	1.6 (1.6)	Evidence is insensitive	Anecdotal evidence for experimental hypothesis
				S: 7‐day point prevalence abstinence	OR 1.07	0.15	> 0.05		OR 1.92^g^	0.3 (0.3)	Evidence for null hypothesis (i.e. no effect)	Moderate evidence for null hypothesis
									OR 1.40α	0.6 (0.6)	Evidence is insensitive	Anecdotal evidence for null hypothesis
									OR 2.40α	0.3 (0.3)	Evidence for null hypothesis (i.e. no effect)	Moderate evidence for null hypothesis
				S: Quit attempt	OR 1.15	0.12	> 0.05		OR 1.92^g^	0.6 (0.6)	Evidence is insensitive	Anecdotal evidence for null hypothesis
									OR 1.40α	1.0 (1.0)	Evidence is insensitive	No evidence
									OR 2.40α	0.4 (0.4)	Evidence is insensitive	Anecdotal evidence for null hypothesis
Rendall‐Mkosi 23	Motivational Interviewing	Minimal intervention	165 women aged 18–44 years at risk of alcohol exposed pregnancy	P: Alcohol exposed pregnancy	OR 0.46	0.35	0.024*	‘A five session motivational interviewing intervention was found to be effective with women at risk of an alcohol‐exposed pregnancy. . . it is noteworthy that the reduction in risk for AEP in this study was mainly due to the improved contraceptive rather than a reduction in risky alcohol use’	OR 1.90^h^	6.5 (6.5)	Evidence for experimental hypothesis (i.e. an effect)	Moderate evidence for experimental hypothesis
									OR 1.36Ω	4.2 (4.2)	Evidence for experimental hypothesis (i.e. an effect)	Moderate evidence for experimental hypothesis
									OR 2.66Ω	6.2 (6.2)	Evidence for experimental hypothesis (i.e. an effect)	Moderate evidence for experimental hypothesis
				S: Risky drinking	OR 0.75	0.53	0.580		OR 0.84^i^	1.1 (1.1)	Evidence is insensitive	Anecdotal evidence for experimental hypothesis
									OR 0.70α	1.1 (1.1)	Evidence is insensitive	Anecdotal evidence for experimental hypothesis
									OR 0.90α	1.1 (1.1)	Evidence is insensitive	Anecdotal evidence for experimental hypothesis
				S: Ineffective contraception	OR 0.51	0.37	0.067	‘At the 12‐month follow‐up, the reduction [in risky drinking] in the MI group (14.75%) was modestly larger when compared to the control group (10.94%), but this difference was also not statistically significant. . the reduction in the proportion of participants who were using ineffective contraception at 12 months was no longer statically significant’	OR 0.63^i^	3.0 (3.0)	Evidence for experimental hypothesis (i.e. an effect)	Moderate evidence for experimental hypothesis
									OR 0.54α	3.2 (3.2)	Evidence for experimental hypothesis (i.e. an effect)	Moderate evidence for experimental hypothesis
									OR 0.74α	2.6 (2.6)	Evidence is insensitive	Anecdotal evidence for experimental hypothesis
Coffin 24	Aripiprazole	Placebo	90 methamphetamine dependent, sexually active adults from the community	P: Methamphetamine use	RR 0.88	0.15	0.410	‘Compared with placebo, apripiprazole did not reduce methamphetamine use significantly among actively dependent adults. . notwithstanding the promising pre‐clinical results suggesting that apripiprazole might be effective at decreasing craving for methamphetamine and reducing it rewarding properties, we found no effect of this medication on methamphetamine use, severity of craving. We also did not evidence that apripiprazole was associated with increased methamphetamine use or rewards, as suggested by some investigators.’	RR 1.12^j^	1.3 (1.3)	Evidence is insensitive	Anecdotal evidence for experimental hypothesis
									RR 1.02α	1.1 (1.1)	Evidence is insensitive	Anecdotal evidence for experimental hypothesis
									RR 1.22α	1.1 (1.1)	Evidence is insensitive	Anecdotal evidence for experimental hypothesis
				S: Adherence – medication event monitoring systems	RR 1.33	0.43	0.310		RR 0.99^k^	1.0 (1.0)	Evidence is insensitive	No evidence
									RR 0.80α	1.2 (1.2)	Evidence is insensitive	Anecdotal evidence for experimental hypothesis
									RR 1.00	0.7 (0.7)	Evidence is insensitive	Anecdotal evidence for null hypothesis
				S: Adherence, self‐reported	RR 0.59	0.49	0.170		RR 1.03^k^	1.1 (1.1)	Evidence is insensitive	Anecdotal evidence for experimental hypothesis
									RR 1.01α	1.0 (1.0)	Evidence is insensitive	No evidence
									RR 1.10α	1.2 (1.2)	Evidence is insensitive	Anecdotal evidence for experimental hypothesis
				S: Number of partners with whom methamphetamines were used	RR 0.38	0.86	0.254		RR 0.45^k^	1.5 (1.5)	Evidence is insensitive	Anecdotal evidence for experimental hypothesis
									RR 0.24Ω	1.4 (1.4)	Evidence is insensitive	Anecdotal evidence for experimental hypothesis
									RR 0.82Ω	1.2 (1.2)	Evidence is insensitive	Anecdotal evidence for experimental hypothesis
				S: Number of sexual partners	RR 0.69	0.46	0.418	‘In the intention‐to‐treat GEE analysis, the risk of testing positive for methamphetamine was similar in the aripiprazole arm compared to the placebo arm. . difference between arms over follow‐up was not significant [in severity of dependence. . After controlling for imbalanced baseline characteristics, sexual risk behaviors declined similarly in the aripiprazole and placebo arms.’	RR 0.20^k^	0.2 (0.2)	Evidence for null hypothesis (i.e. no effect)	Strong evidence for null hypothesis
									RR 0.04Ω	0.1 (0.1)	Evidence for null hypothesis (i.e. no effect)	Strong evidence for null hypothesis
									RR 0.93Ω	0.9 (0.9)	Evidence is insensitive	Anecdotal evidence for null hypothesis
				S: Episodes of anal and/or vaginal sex with sero‐discordant partners	RR 0.42	0.65	0.190		RR 0.31^k^	1.7 (1.7)	Evidence is insensitive	Anecdotal evidence for experimental hypothesis
									RR 0.14Ω	1.3 (1.3)	Evidence is insensitive	Anecdotal evidence for experimental hypothesis
									RR 0.66Ω	1.7 (1.7)	Evidence is insensitive	Anecdotal evidence for experimental hypothesis
				S: Episodes of unprotected anal and/or vaginal sex wth sero‐discordant partners	RR 0.61	0.98	0.612		RR 0.34^k^	0.9 (0.9)	Evidence is insensitive	Anecdotal evidence for null hypothesis
									RR 0.17Ω	0.7 (0.7)	Evidence is insensitive	Anecdotal evidence for null hypothesis
									RR 0.70Ω	1.1 (1.1)	Evidence is insensitive	Anecdotal evidence for experimental hypothesis
				S: Episodes of insertive unprotected anal sex with sero‐discordant partners	RR 0.54	0.72	0.385		RR 0.29^k^	1.0 (1.0)	Evidence is insensitive	Anecdotal evidence for experimental hypothesis
									RR 0.14Ω	0.8 (0.8)	Evidence is insensitive	Anecdotal evidence for null hypothesis
									RR 0.58Ω	1.3 (1.3)	Evidence is insensitive	Anecdotal evidence for experimental hypothesis
				S: Episodes of receptive unprotected anal and/or vaginal sex with sero‐discordant partners	RR 0.02	1.32	0.007**		RR 0.27^k^	12.0 (12.0)	Evidence for experimental hypothesis (i.e. an effect)	Strong evidence for experimental hypothesis
									RR 0.05Ω	30.9 (30.9)	Evidence for experimental hypothesis (i.e. an effect)	Very strong evidence for experimental hypothesis
									RR 0.49Ω	4.4 (4.4)	Evidence for experimental hypothesis (i.e. an effect)	Moderate evidence for experimental hypothesis
				S: Methamphetamine craving	MD 6.8	7.65	0.380		MD 35^k^	0.5 (0.5)	Evidence is insensitive	Anecdotal evidence for null hypothesis
									MD 8Ω	1.3 (1.3)	Evidence is insensitive	Anecdotal evidence for experimental hypothesis
									MD 62Ω	0.3 (0.3)	Evidence for null hypothesis (i.e. no effect)	Strong evidence for null hypothesis
				S: Severity of dependence	MD −0.04	0.85	0.960		MD 2.00^l^	0.4 (0.4)	Evidence is insensitive	Anecdotal evidence for null hypothesis
									MD 1.00α	0.7 (0.7)	Evidence is insensitive	Anecdotal evidence for null hypothesis
									MD 3.00α	0.3 (0.3)	Evidence for null hypothesis (i.e. no effect)	Strong evidence for null hypothesis
				S: Depression	MD 1.47	2.19	0.500		MD 2.00^l^	1.1 (1.1)	Evidence is insensitive	Anecdotal evidence for experimental hypothesis
									MD 1.00α	1.2 (1.2)	Evidence is insensitive	Anecdotal evidence for experimental hypothesis
									MD 3.00α	1.0 (1.0)	Evidence is insensitive	Anecdotal evidence for experimental hypothesis
Gilbert 25	Tailored cessation on advice reports, including levels of reading ability	Generic self‐help booklet	58 66 current cigarette smokers aged 18–65 years, identified from general practitioner records	P: Prolonged abstinence for 3 months	OR 1.18	0.13	0.184	‘ESCAPE. . appears to increase the rate at which smokers try to stop, but if there is an effect on prolonged abstinence it is small… Quit rates for the primary outcome of three months of prolonged abstinence were not significantly different between study groups. Thus, the intervention showed no effect. Quit rates in a number of different outcome measures of abstinence also showed no significant effect. However, all outcome measures showed a non‐significant trend towards more abstinence in the intervention group’	OR 1.42^m^	1.3 (1.3)	Evidence is insensitive	Anecdotal evidence for experimental hypothesis
									OR 1.21Ω	1.7 (1.7)	Evidence is insensitive	Anecdotal evidence for experimental hypothesis
									OR 1.68Ω	0.9 (0.9)	Evidence is insensitive	Anecdotal evidence for null hypothesis
				S: Prolonged abstinence for 1 month	OR 1.17	0.11	0.130		OR 1.42^m^	1.5 (1.5)	Evidence is insensitive	Anecdotal evidence for experimental hypothesis
									OR 1.21Ω	2.0 (2.0)	Evidence is insensitive	Anecdotal evidence for experimental hypothesis
									OR 1.68Ω	1.1 (1.1)	Evidence is insensitive	Anecdotal evidence for experimental hypothesis
				S: 7‐day point prevalence abstinence	OR 1.11	0.10	0.307		OR 1.42^m^	0.8 (0.8)	Evidence is insensitive	Anecdotal evidence for null hypothesis
									OR 1.21Ω	1.1 (1.1)	Evidence is insensitive	Anecdotal evidence for experimental hypothesis
									OR 1.68Ω	0.5 (0.5)	Evidence is insensitive	Anecdotal evidence for null hypothesis
				S: 24‐hour point prevalence abstinence	OR 1.15	0.09	0.131		OR 1.42^m^	1.4 (1.4)	Evidence is insensitive	Anecdotal evidence for experimental hypothesis
									OR 1.21Ω	2.1 (2.1)	Evidence is insensitive	Anecdotal evidence for experimental hypothesis
									OR 1.68Ω	1.0 (1.0)	Evidence is insensitive	Anecdotal evidence for experimental hypothesis
				S: Quit attempt	OR 1.11	0.06	0.074	‘The difference [in 3 month prolonged abstinence] was not significant. . No significant differences were found between the intervention and control groups on shorter periods or on point‐prevalence measures of abstinence’.	OR 1.42^m^	1.4 (1.4)	Evidence is insensitive	Anecdotal evidence for experimental hypothesis
									OR 1.21Ω	2.3 (2.3)	Evidence is insensitive	Anecdotal evidence for experimental hypothesis
									OR 1.68Ω	1.0 (1.0)	Evidence is insensitive	Anecdotal evidence for experimental hypothesis
Alessi 26	Compensation for video recording alcohol breath tests using a cell phone and contingency management with escalating vouchers for on‐time alcohol‐negative tests.	Compensation for video recording alcohol breath tests using a cell phone	30 adults who drank frequently but were not physiologically dependent	P: Negative breath sample	MD 20.20	5.74	< 0.001***	‘Cellphone technology may be useful for extending contingency management to treatment for alcohol problems’	MD 8.00^n^	69.8 (69.9)	Evidence for experimental hypothesis (i.e. an effect)	Very strong evidence for experimental hypothesis
									MD 5.00α	21.7 (21.7)	Evidence for experimental hypothesis (i.e. an effect)	Strong evidence for experimental hypothesis
									MD 12.00α	134.1 (134.2)	Evidence for experimental hypothesis (i.e. an effect)	Extreme evidence for experimental hypothesis
				S: Longest duration of negative samples	MD 10.90	3.52	< 0.001***	No mention of results > 0.05	MD 2.00^n^	5.3 (5.3)	Evidence for experimental hypothesis (i.e. an effect)	Moderate evidence for experimental hypothesis
									MD 1.00α	2.2 (2.2)	Evidence is insensitive	Moderate evidence for experimental hypothesis
									MD 3.00α	11.2 (11.2)	Evidence for experimental hypothesis (i.e. an effect)	Strong evidence for experimental hypothesis
				S: Days of drinking	MD −11.00	3.48	< 0.001***		MD 3.71^o^	19.5 (19.5)	Evidence for experimental hypothesis (i.e. an effect)	Strong evidence for experimental hypothesis
									MD 1.00α	2.3 (2.3)	Evidence is insensitive	Moderate evidence for experimental hypothesis
									MD 7.00α	49.4 (49.4)	Evidence for experimental hypothesis (i.e. an effect)	Very strong evidence for experimental hypothesis
				S: Drinks per drinking day	MD −0.80	0.83	0.350		MD 1.20^o^	1.2 (1.2)	Evidence is insensitive	Anecdotal evidence for experimental hypothesis
									MD 0.5α	1.3 (1.3)	Evidence is insensitive	Anecdotal evidence for experimental hypothesis
									MD 1.90α	1.0 (1.0)	Evidence is insensitive	No evidence
				S: Addiction Severity Index	MD −0.09	0.03	0.010**		MD 0.10^n^	41.3 (41.3)	Evidence for experimental hypothesis (i.e. an effect)	Very strong evidence for experimental hypothesis
									MD 0.01α	2.6 (2.6)	Evidence is insensitive	Anecdotal evidence for experimental hypothesis
									MD 0.20α	28.0 (28.0)	Evidence for experimental hypothesis (i.e. an effect)	Very strong evidence for experimental hypothesis
				S: Drinker Inventory of Consequences	MD −0.80	0.23	< 0.001***		MD 1.00^p^	120.0 (120.0)	Evidence for experimental hypothesis (i.e. an effect)	Extreme evidence for experimental hypothesis
									MD 0.2Ω	18.1 (18.1)	Evidence for experimental hypothesis (i.e. an effect)	Strong evidence for experimental hypothesis
									MD 1.8Ω	83.4 (83.4)	Evidence for experimental hypothesis (i.e. an effect)	Very strong evidence for experimental hypothesis
Richmond 27	Nortriptyline added to multi‐component smoking cessation intervention (included nicotine replacement therapy and cognitive behavioural therapy)	Placebo added to multi‐component smoking cessation intervention (included nicotine replacement therapy and cognitive behavioural therapy)	425 male prisoners	P: Continuous abstinence	OR 0.98	0.30	> 0.05	‘Adding nortriptyline to a smoking cessation treatment package consisting of behavioural support and nicotine replacement therapy does not appear to improve long‐term abstinence rates in male prisoners. . In this study, we found no significant difference in an intention‐to‐treat analysis between the two study groups, suggesting that the additional use of NOR does not enhance quit rates for tobacco in the longer term’	OR 1.21^q^	0.9 (0.9)	Evidence is insensitive	Moderate evidence for null hypothesis
									OR 1.01Ω	1.0 (1.0)	Evidence is insensitive	No evidence
									OR 1.55Ω	0.6 (0.6)	Evidence is insensitive	Moderate evidence for null hypothesis
				P: Point prevalence abstinence	OR 0.81	0.29	> 0.05		OR 1.21^q^	1.1 (1.1)	Evidence is insensitive	Anecdotal evidence for experimental hypothesis
									OR 1.01Ω	1.0 (1.0)	Evidence is insensitive	No evidence
									OR 1.55Ω	1.0 (1.0)	Evidence is insensitive	No evidence
				S: Smoking reduction (>50% reduction in cigarette consumption)	OR 0.75	0.26	> 0.05	‘Based on an intention‐to‐treat analysis and cut‐off point for CO of ≤ 10 p.p.m, continuous abstinence between the treatment and comparison groups were not statistically different at 3 months. . point‐prevalence abstinence, using the ≤ 5 p.p.m. cut‐off between the treatment and control groups, was also not statistically significant different at three months’.	OR 0.43^q^	0.9 (0.9)	Evidence is insensitive	Moderate evidence for null hypothesis
									OR 0.12Ω	0.4 (0.4)	Evidence is insensitive	Moderate evidence for null hypothesis
									OR 0.99Ω	1.0 (1.0)	Evidence is insensitive	No evidence
Levin 28	Venlafaxine‐extended release	Placebo	103 cannabis dependent adults	P: Two‐week abstinence	OR 0.23	0.52	< 0.001***	‘For depressed, cannabis‐dependent patients, venlafaxine‐extended release does not appear to be effective at reducing depression and may lead to an increase in cannabis use’	OR 0.80^r^	2.9 (2.9)	Evidence for experimental hypothesis (i.e. an effect)	Moderate evidence for experimental hypothesis
									OR 0.70α	5.5 (5.5)	Evidence for experimental hypothesis (i.e. an effect)	Moderate evidence for experimental hypothesis
									OR 0.90α	1.6 (1.6)	Evidence is insensitive	Anecdotal evidence for experimental hypothesis
				P: 50% reduction in depressive symptoms (Hamilton Depression rating scale)	OR 0.75	0.42	0.510		OR 1.43^s^	1.1 (1.1)	Evidence is insensitive	Anecdotal evidence for experimental hypothesis
									OR 1.20α	1.1 (1.1)	Evidence is insensitive	Anecdotal evidence for experimental hypothesis
									OR 1.60α	1.1 (1.1)	Evidence is insensitive	Anecdotal evidence for experimental hypothesis
				S: THC urine levels	MD 964	320.27	< 0.001***	‘No significant effect of treatment and no significant effect of baseline HAMD on 50% reduction of HAMD’.	MD 137.3^t^	3.3 (3.3)	Evidence for experimental hypothesis (i.e. an effect)	Moderate evidence for experimental hypothesis
									MD 100α	2.3 (2.3)	Evidence is insensitive	Anecdotal evidence for experimental hypothesis
									MD 300α	11.9 (11.9)	Evidence for experimental hypothesis (i.e. an effect)	Strong evidence for experimental hypothesis
				S: Use in grams	MD 2.67	4.72	0.320		MD 0.45^u^	1.0 (1.0)	Evidence is insensitive	No evidence
									MD 0.02α	1.0 (1.0)	Evidence is insensitive	No evidence
									MD 0.88α	1.1 (1.1)	Evidence is insensitive	Anecdotal evidence for experimental hypothesis
Okuyemi 29	Motivational interviewing and nicotine patch	Nicotine patch and brief advice to quit	430 homeless smokers	P: 7‐day point prevalence abstinence	OR 1.33	0.21	0.170	‘Adding motivation interviewing counselling for nicotine patch did not increase smoking rate significantly at 26‐week follow‐up for homeless smokers. . MI did not improve adherence measures among participants who received MI.’	OR 1.35^v^	1.8 (1.8)	Evidence is insensitive	Anecdotal evidence for experimental hypothesis
									OR 1.02Ω	1.1 (1.1)	Evidence is insensitive	Anecdotal evidence for experimental hypothesis
									OR 1.78Ω	1.4 (1.4)	Evidence is insensitive	Anecdotal evidence for experimental hypothesis
				S: Motivation to adhere	MD 1.4	0.49	0.080		MD 4.97^w^	11.2 (11.2)	Evidence for experimental hypothesis (i.e. an effect)	Strong evidence for experimental hypothesis
									MD 1.19 Ω	25.0 (25.0)	Evidence for experimental hypothesis (i.e. an effect)	Strong evidence for experimental hypothesis
									MD 8.75Ω	6.6 (6.6)	Evidence for experimental hypothesis (i.e. an effect)	Moderate evidence for experimental hypothesis
				S:Self‐efficacy to adhere	MD 2.5	3.12	0.220	‘Motivation for adherence scores at week 6 were marginally higher for participants in the intervention group than those in the control group. . There were no differences between study groups in the proportion of participants who had their nicotine patches on at various study visits’.	MD 4.97 w	1.0 (1.0)	Evidence is insensitive	Anecdotal evidence for experimental hypothesis
									MD 1.19Ω	1.2 (1.2)	Evidence is insensitive	Anecdotal evidence for experimental hypothesis
									MD 8.75Ω	0.7 (0.7)	Evidence is insensitive	Anecdotal evidence for null hypothesis
				S: Nicotine patch use	OR 1.0	0.20	0.970		OR 1.14^z^	0.8 (0.8)	Evidence is insensitive	Moderate evidence for null hypothesis
									OR 1.02α	1.0 (1.0)	Evidence is insensitive	No evidence
									OR 1.28α	0.6 (0.6)	Evidence is insensitive	Moderate evidence for null hypothesis
Gustafson 30	Interest circle calls	No intervention	201 clinics	P: Waiting‐time (mean days between first contact and first treatment)	MD −0.24	2.12	0.911	‘When trying to improve the effectiveness of addiction treatment services, clinic‐level coaching appears to help improve waiting‐time and number of new patients while other components of improvement collaboratives (interest circle calls and learning sessions) do not seem to add further value’	MD 10.6^y^	0.2 (0.2)	Evidence for null hypothesis (i.e. no effect)	Strong evidence for null hypothesis
									MD 15α	0.2 (0.2)	Evidence for null hypothesis (i.e. no effect)	Strong evidence for null hypothesis
									MD 5α	0.4 (0.4)	Evidence is insensitive	Moderate evidence for null hypothesis
				P: Retention (percentage of patients retained from first to fourth treatment session)	MD −0.003	0.03	0.912		MD 7.5^y^	0.01 (0.01)	Evidence for null hypothesis (i.e. no effect)	Very strong evidence for null hypothesis
									MD 10α	0.00 (0.00)	Evidence for null hypothesis (i.e. no effect)	Extreme evidence for null hypothesis
									MD 5α	0.01 (0.01)	Evidence for null hypothesis (i.e. no effect)	Very strong evidence for null hypothesis
				P: Annual number of new patients	MD −0.04	0.04	0.369	‘Learning sessions had a modest waiting time reduction while interest circle calls had a slight increase, but these two groups’ changes were not statistically significant. . None of the groups showed significant improvement in retention for the 6‐month intervention period (Table 3a), or the entire intervention and sustainability period (Table 3b), and there were no significant differences between groups’	MD 14.2^y^	0.01 (0.01)	Evidence for null hypothesis (i.e. no effect)	Very strong evidence for null hypothesis
									MD 20α	0.00 (0.00)	Evidence for null hypothesis (i.e. no effect)	Extreme evidence for null hypothesis
									MD 10α	0.01 (0.00)	Evidence for null hypothesis (i.e. no effect)	Very strong evidence for null hypothesis
	Coaching	No intervention		P: Waiting‐time (mean days between first contact and first treatment)	MD 4.86	1.95	0.013*		MD 10.6^y^	7.2 (7.2)	Evidence for experimental hypothesis (i.e. an effect)	Moderate evidence for experimental hypothesis
									MD 15α	5.4 (5.4)	Evidence for experimental hypothesis (i.e. an effect)	Moderate evidence for experimental hypothesis
									MD 5α	10.7 (10.7)	Evidence for experimental hypothesis (i.e. an effect	Strong evidence for experimental hypothesis
				P: Retention (percentage of patients retained from first to fourth treatment session)	MD 0.035	0.02	0.118		MD 7.5^y^	0.0 (0.0)	Evidence for null hypothesis (i.e. no effect)	Extreme evidence for null hypothesis
									MD 10α	0.0 (0.0)	Evidence for null hypothesis (i.e. no effect)	Extreme evidence for null hypothesis
									MD 5α	0.0 (0.0)	Evidence for null hypothesis (i.e. no effect)	Extreme evidence for null hypothesis
				P: Annual number of new patients	MD 0.20	0.09	0.028*		MD 0.14^y^	6.0 (6.0)	Evidence for experimental hypothesis (i.e. an effect)	Moderate evidence for experimental hypothesis
									MD 0.20α	6.3 (6.3)	Evidence for experimental hypothesis (i.e. an effect)	Moderate evidence for experimental hypothesis
									MD 0.10α	5.0 (5.0)	Evidence for experimental hypothesis (i.e. an effect	Moderate evidence for experimental hypothesis
	Learning sessions	No intervention		P: Waiting‐time (mean days between first contact and first treatment)	MD 3.14	1.93	0.103		MD 10.6^y^	1.2 (1.2)	Evidence is insensitive	Anecdotal evidence for experimental hypothesis
									MD 15α	0.9 (0.9)	Evidence is insensitive	Anecdotal evidence for null hypothesis
									MD 5α	2.1 (2.1)	Evidence is insensitive	Anecdotal evidence for experimental hypothesis
				P: Retention (percentage of patients retained from first to fourth treatment session)	MD −0.003	0.02	0.899		MD 7.5^y^	0.00 (0.00)	Evidence for null hypothesis (i.e. no effect)	Extreme evidence for null hypothesis
									MD 10α	0.00 (0.00)	Evidence for null hypothesis (i.e. no effect)	Extreme evidence for null hypothesis
									MD 5α	0.00 (0.00)	Evidence for null hypothesis (i.e. no effect)	Extreme evidence for null hypothesis
				P: Annual number of new patients	MD −0.001	0.07	0.982		MD 14.2^y^	0.00 (0.00)	Evidence for null hypothesis (i.e. no effect)	Extreme evidence for null hypothesis
									MD 20α	0.00 (0.00)	Evidence for null hypothesis (i.e. no effect)	Extreme evidence for null hypothesis
									MD 10α	0.01 (0.01)	Evidence for null hypothesis (i.e. no effect)	Very strong evidence for null hypothesis
	Combination	No intervention		P: Waiting‐time (mean days between first contact and first treatment)	MD 6.16	1.97	0.002**		MD 10.6^y^	41.2 (41.2)	Evidence for experimental hypothesis (i.e. an effect)	Very strong evidence for experimental hypothesis
									MD 15α	31.8 (31.8)	Evidence for experimental hypothesis (i.e. an effect)	Very strong evidence for experimental hypothesis
									MD 5α	50.4 (50.4)	Evidence for experimental hypothesis (i.e. an effect	Very strong evidence for experimental hypothesis
				P: Retention (percentage of patients retained from first to fourth treatment session)	MD −0.003	0.02	0.891		MD 7.5^y^	0.00 (0.00)	Evidence for null hypothesis (i.e. no effect)	Extreme evidence for null hypothesis
									MD 10α	0.00 (0.00)	Evidence for null hypothesis (i.e. no effect)	Extreme evidence for null hypothesis
									MD 5α	0.00 (0.00)	Evidence for null hypothesis (i.e. no effect)	Extreme evidence for null hypothesis
				P: Annual number of new patients	MD 0.09	0.04	0.029*		MD 0.14^y^	5.6 (5.6)	Evidence for experimental hypothesis (i.e. an effect)	Moderate evidence for experimental hypothesis
									MD 0.20α	4.4 (4.4)	Evidence for experimental hypothesis (i.e. an effect)	Moderate evidence for experimental hypothesis
									MD 0.10α	6.5 (6.5)	Evidence for experimental hypothesis (i.e. an effect	Moderate evidence for experimental hypothesis

P = primary outcome; S = secondary outcome;

* significant at *P* < 0.05;

** significant at *P* < 0.01;

*** significant at *P* < 0.001;

RaR = rate ratio; RR = relative risk; OR = odds ratio; MD = mean difference;

Ω range of population SD reflects the CI of the expected effect size;

α range of population SD based on opinion on a viable effect; a one‐directional relationship was assumed in all instances; Based on:

a 31;

b 32;

c 33;

d 34;

e 35;

f 36;

g values specified in the sample size calculation;

h 37;

i 38;

j 39;

k 40;

l 41;

m 42;

n 43;

o 44;

p 45;

q 46;

r 47;

s 48;

t 49;

u 50;

v 51;

w 52;

z 53;

y values specified in the sample size calculation.

HAMD = Hamilton Rating Scale for Depression; p.p.m. = parts per million.

When specifying the predicted effect, we used a ‘half normal distribution’ whose peak was at 0 (no effect) and extending upwards with a SD equal to the expected effect size. This represents a hypothesis that the intervention had at least some positive effect, with the effect being more likely to be smaller than larger. This is a conservative approach to prediction. Another approach would be to specify the hypothesis as a uniform distribution between 0 (or a minimally clinically significant value) and a plausible upper bound. Given that none of the authors of the studies reviewed indicated what they considered to be a clinically meaningful effect or a plausible upper bound for the effect size, we took the conservative approach.

## Results

Of the 12 studies, 55 non‐significant effects and 20 significant effects were reported. For each of these, three Bayes factors were calculated: one based on an expected population SD (identified from previous studies) and two based on a range of values around the expected population SD (identified from previous studies or based on expert opinion). Thus, a total of 75 Bayes factors were calculated in the main analysis and 150 Bayes factors were derived in the sensitivity analysis (see Table 2).

Fifty‐six per cent (*n* = 42) of the Bayes factors were between ⅓ and 3; 14.7% (*n* = 11) were < ⅓ and 29.3% (*n* = 22) were > 3. When considering only the non‐significant findings (*n* = 55), 20.0% (*n* = 11) of Bayes factors were < ⅓ and 3.6% (*n* = 2) were > 3. The other 76.4% (*n* = 42) of Bayes factors were between ⅓ and 3. Of these, 26 were in the direction of there being an effect (Bayes factor > 1 and < 3); 12 tended to favour the hypothesis of no effect (Bayes factor < 1 and > ⅓); and for four there was no evidence either way (Bayes factor = 1).

In sensitivity analyses, 13.3% of Bayes factors were < ⅓ (*n* = 20), 62.7% (*n* = 94) were between ⅓ and 3 and 24.0% (*n* = 36) were >3, showing good consistency with the main results.

Authors either decided not to discuss results where *P* > 0.05, to report them as non‐significant and/or to state that no association was found. Good concordance was noted between the online calculator 8 and the adapted R code 9, except for those Bayes factors that indicated extreme evidence for the experimental hypothesis.

## Discussion

Only ⅕ of all non‐significant findings provided support for the hypothesis of no effect, while nearly ⅔ of the Bayes factors indicated data insensitivity. Thus, reporting ‘no difference’ between conditions or lack of associations was appropriate for only a small number of papers. A minority of Bayes factors for the non‐significant effects also supported the experimental hypothesis; this tended to occur with *P*‐values close to statistical significance.

The development of online calculators and R code 9, 10 means that researchers in the addiction field can calculate Bayes factors easily to include as an adjunct to traditional frequentist results. The requirement to specify the experimental hypothesis means that scientific judgement is needed. This is a common criticism of Bayesian type methods 17, but it can also be a potential strength, because it forces researchers to be specific about what it is they are testing. Moreover, if there are differences of view about what may be plausible values of the effect size, it is a simple matter to conduct sensitivity analyses to assess what, if any, difference this makes. As a rule of thumb, if one is interested in a clinically relevant range then the uniform distribution can be specified; alternatively, one can use a half‐normal distribution with the peak at 0 if one is interested in any effect at all and has little confidence in the probable value. To prevent researcher bias, pre‐specified analysis plans may be published which detail the method which will be used to calculate Bayes factors, the cut‐off values for interpretation and the plausible effect size which is expected.

The findings of this review show that researchers should avoid the use of terms such as ‘no difference’ or ‘lack of associations’ for *P*‐values > 0.05, unless a Bayes factor < 0.3 is also found. Otherwise null findings should be framed as ‘the findings were inconclusive as to whether or not a difference/association was present’, or some similar wording. This is now encouraged practice by the *Addiction* journal 1. Researchers may also wish to use Bayes factors in order to quantify the evidence for the experimental hypothesis (i.e. moderate, strong, very strong and extreme) and/or use such a calculation as a stopping rule for data collection 18. For ethical and perhaps financial reasons interim analyses are often planned for randomized trials, with early stopping occurring if there is demonstrated efficacy, the intervention is harmful or there is no beneficial effect. *P*‐values cannot inform about us about the latter; in contrast, a Bayes factor indicating data insensitivity would suggest further recruitment, while a Bayes factor indicating evidence for the null hypothesis may point towards early termination.

Note that the methods used to derive Bayes factors in this paper did not cover all the possibilities. More advanced Bayesian hierarchical modelling (BHM) 11, implemented in R and winBUGS, allows a wider range of distributions, e.g. gamma, Poisson, binomial and negative binomial.

## Declaration of interests

E.B. has received unrestricted funding from Pfizer. R.W. undertakes consultancy and research for and receives travel funds and hospitality from manufacturers of smoking cessation medications but does not, and will not, take funds from EC manufacturers or the tobacco industry. R.W. is an advisor to the National Centre for Smoking Cessation Z.D. has no conflicts of interest to declare.

## Supporting information


**Appendix S1** Example: calculating a Bayes Factor.

Supporting info item
