# Supplementary material for: Using Bayes factors for testing hypotheses about intervention effectiveness in addictions research
Source: Addiction. 2016 Aug 10;111(12):2230–47. doi: 10.1111/add.13501 (PMC5111611; doi:10.1111/add.13501)

**Supplementary Appendix 1**

We present here a worked example to show how to use Dienes’ online calculator to obtain Bayes Factors.

http://www.lifesci.sussex.ac.uk/home/Zoltan_Dienes/inference/bayes_factor.swf

Okuyemi et al (29) were interested in the effects of adding motivation interviewing (MI) counselling to the nicotine patch for smoking cessation among homeless smokers. They conducted a randomised controlled trial, whereby 430 participants were randomised to the intervention group or a control group. Verified seven-day abstinence rates at week 26 for the intervention group was non-significantly higher than for the control group (OR 1.33; 95% CI=0.88, 2.02; p= 0.17). They concluded that “Adding motivational interviewing counselling to nicotine patch did not significantly increase smoking rate at 26-week follow-up for homeless smokers”.

To calculate the Bayes Factor the odds ratio first needs to be transformed using a natural logarithmic transformation:

LN(1.33)= 0.29 (2 dp)

and the standard error calculated as:

[LN(2.02)-LN(0.88)]/3.92= 0.21 (2 dp)

We must then decide between three possible distributions to represent the predictions of the theory: uniform, normal or half-normal. If we can only specify a plausible maximum effect we should use the uniform distribution. In contrast, if a plausible predicted effect size can be specified we should opt for a normal or half-normal distribution. The choice between these depends on whether a directional hypothesis can be made, with the latter assuming a one-tailed test. In our example, a half-normal distribution is used as we hypothesize a positive impact of the intervention and can easily derive a predicted value from (51), which was a comprehensive meta-analysis of the use of MI for smoking cessation. This identified an OR for long-term follow-up of 1.35 (95%CI 1.02 to 1.78), which translates to a log odds ratio of 0.30.

We can now calculate our Bayes Factor. First mark the box ‘no’ next to ‘Is the distribution of p(population value|theory) uniform?’


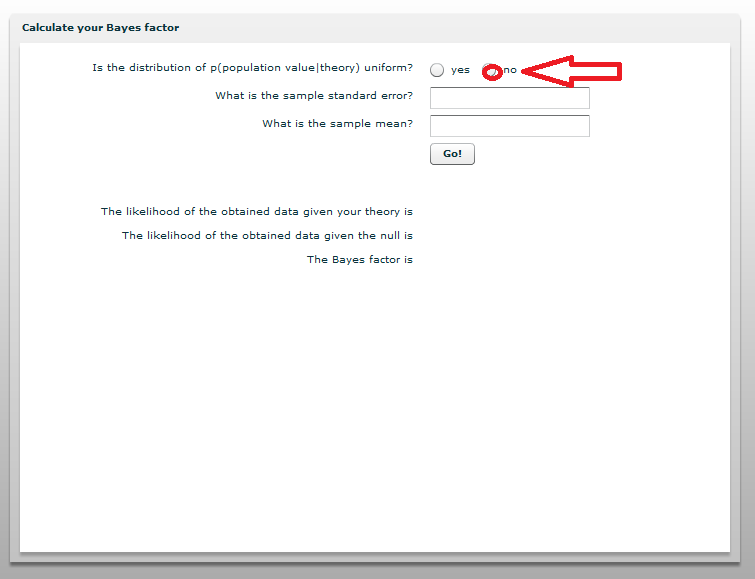


You will then see the following screen with three new boxes. These ask for the mean, standard deviation and number of tails (of a normal). As we are using a half-normal we set mean to 0 (Note: half-normal distribution has a mode of 0), SD to our plausible expected value (Note: this scales the half-normal distribution’s rate of drop) and tails to 1. We must also enter the standard error and mean of our sample. Then click "Go".


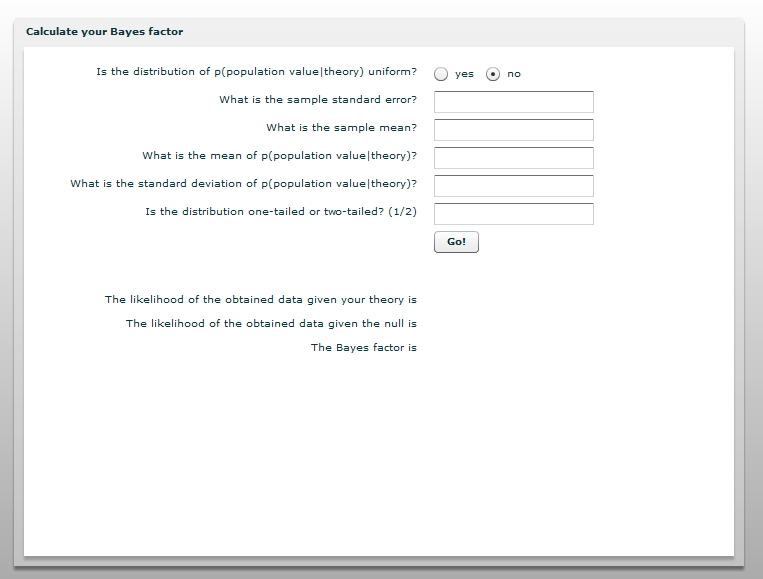


This gives us a Bayes Factor of 1.82, indicating that the data favour the experimental hypothesis but not to a sufficient degree and are thus ‘insensitive’.


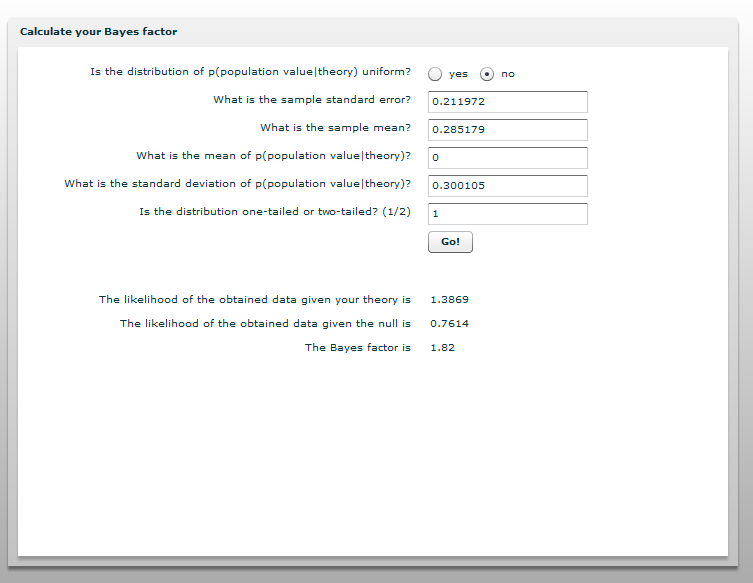

Supplement: Supplementary file 1 — Supporting info item [file ADD-111-2230-s001.docx]
